# Supplementary material for: Antihypertensive, cardio- and neuro-protective effects of Tenebrio molitor (Coleoptera: Tenebrionidae) defatted larvae in spontaneously hypertensive rats
Source: PLoS One. 2020 May 29;15(5):e0233788. doi: 10.1371/journal.pone.0233788 (PMC7259609; doi:10.1371/journal.pone.0233788)
Supplement: S1 File — Defatted larvae used in the paper were obtained extracting twice with ethanol (99.5%) for 1 h as reported by Zhao et al. [45]. The extraction was accomplished using a solvent to material ratio of 5 mL/g, at 40°C for 1 h. For the dry matter determination 3 g exactly weighed ground larvae were dried overnight at 104°C [46]. Fat content was determined by Soxhlet extraction method using petroleum spirit for six hours starting from 5 g of sample. The solvent was removed at 40° under rotary evaporator and extracted fat weighted after the flask reaching constant weigh after heating at 104°C. Crude protein were determined with the Kjeldahl method using a protein-to-nitrogen conversion factor of 6.25 [2]. Ash were determined by weighting after incineration of 2 g of sample at 550°C for 3 h and cooling in a desiccator [2]. Each assay was run at least in triplicate. The analysis gave in the starting material dry matter (95.8±0.08%), fat content (32.7±0.84%), crude proteins (50.8±0.72%) and ash (4.8±0.09%), extracted fat (33.8±0.92%) and crude protein content in the final product (77.5±0.59%). These values were superimposable to those of Zhao et al. [45]. Nutritional value of TM protein as well as the in vitro digestibility was already accomplished [47]. Proteins extracted as water-soluble fraction (supernatant) or as water-insoluble fraction (pellet) were characterized by SD-PAGE and LC-MS/MS [48]. The most abundant in the supernatant were hemolymph protein (~12 kDa), alpha-amylase (~50 kDa, a putative allergen), and muscle proteins (e.g. actin 30–50 kDa) in the pellet fraction. Also the amino acid pattern and the essential amino acid index were already described [49] and [doi 10.3844/ajabssp.2009.319.331], showing that TM contain all the essential amino acid in the amount necessary to human [50]. The sum of total amount of amino acids (TAA) per g crude protein of TM was 910 mg/g, while the calculated essential amino acid index (EAAI) was slightly higher than that of soybean, [file pone.0233788.s001.docx]

# Supporting Information

**S1 File. Defatting of T. molitor larvae*.*** Defatted larvae used in the paper were obtained extracting twice with ethanol (99.5 %) for 1 h as reported by Zhao et al. [45]. The extraction was accomplished using a solvent to material ratio of 5 mL/g, at 40°C for 1 h. For the dry matter determination 3 g exactly weighed ground larvae were dried overnight at 104°C [46]. Fat content was determined by Soxhlet extraction method using petroleum spirit for six hours starting from 5 g of sample. The solvent was removed at 40° under rotary evaporator and extracted fat weighted after the flask reaching constant weigh after heating at 104°C. Crude protein were determined with the Kjeldahl method using a protein-to-nitrogen conversion factor of 6.25 [2]. Ash were determined by weighting after incineration of 2 g of sample at 550°C for 3 h and cooling in a desiccator [2]. Each assay was run at least in triplicate. The analysis gave in the starting material dry matter (95.8±0.08%), fat content (32.7±0.84%), crude proteins (50.8±0.72%) and ash (4.8±0.09%), extracted fat (33.8±0.92%) and crude protein content in the final product (77.5±0.59%). These values were superimposable to those of Zhao et al. [45].

Nutritional value of TM protein as well as the *in vitro* digestibility was already accomplished [47]. Proteins extracted as water-soluble fraction (supernatant) or as water-insoluble fraction (pellet) were characterized by SD-PAGE and LC-MS/MS [48]. The most abundant in the supernatant were hemolymph protein (~12 kDa), alpha-amylase (~50 kDa, a putative allergen), and muscle proteins (*e.g.* actin 30–50 kDa) in the pellet fraction.

Also the amino acid pattern and the essential amino acid index were already described [49] and [doi 10.3844/ajabssp.2009.319.331], showing that TM contain all the essential amino acid in the amount necessary to human [50]. The sum of total amount of amino acids (TAA) per g crude protein of TM was 910 mg/g, while the calculated essential amino acid index (EAAI) was slightly higher than that of soybean, but lower than that of casein. Moreover, the protein digestibility-corrected amino acid score (PDCAAS) of defatted TM was 0.80, further confirming that it has the ability to meet human dietary requirements for specific amino acids (0.79-0.82 for PDCAAS) [doi.org/10.3920/JIFF2018.0048].

Toxicological aspects such as subchronic toxicity of TM diet, including potential hypersensitivity, after orally administration at dose up to 3000 mg/kg/day for 90 days, was already assessed in both sexes of Sprague-Dawley rats [21]. TM was also proven to be non-genotoxic ([51], erratum in [52]).
