# Supplementary material for: Antihypertensive, cardio- and neuro-protective effects of Tenebrio molitor (Coleoptera: Tenebrionidae) defatted larvae in spontaneously hypertensive rats
Source: PLoS One. 2020 May 29;15(5):e0233788. doi: 10.1371/journal.pone.0233788 (PMC7259609; doi:10.1371/journal.pone.0233788)
Supplement: S2 File — SHR and WKY rats were fully anaesthetized by intraperitoneal injection of 15 mg/kg Zoletil 100® (Virbac Srl, Milano, Italy) and 4 mg/kg Xylor® (Bio 98, San Lazzaro, Italy). Zoletil 100® is a 1:1 mixture of tiletamine (dissociative anaesthetic acting as NMDA receptor antagonist) and zolazepam (benzodiazepine). Xylor® is composed by the α2 adrenergic receptor agonist xylazine endowed with sedation and muscle relaxation activities. (DOCX) [file pone.0233788.s002.docx]

**Supporting Information**

**S2 File. Details of animal anaesthesia.** SHR and WKY rats were fully anaesthetized by intraperitoneal injection of 15 mg/kg Zoletil 100® (Virbac Srl, Milano, Italy) and 4 mg/kg Xylor® (Bio 98, San Lazzaro, Italy). Zoletil 100® is a 1:1 mixture of tiletamine (dissociative anaesthetic acting as NMDA receptor antagonist) and zolazepam (benzodiazepine). Xylor® is composed by the α_2_ adrenergic receptor agonist xylazine endowed with sedation and muscle relaxation activities.
