# Supplementary material for: Antihypertensive, cardio- and neuro-protective effects of Tenebrio molitor (Coleoptera: Tenebrionidae) defatted larvae in spontaneously hypertensive rats
Source: PLoS One. 2020 May 29;15(5):e0233788. doi: 10.1371/journal.pone.0233788 (PMC7259609; doi:10.1371/journal.pone.0233788)
Supplement: S3 File — (DOCX) [file pone.0233788.s003.docx]

**Supporting Information**

**S3 File.** **RP-HPLC determination of plasma and brain ACE and its inhibitory activity**

***Determination of ACE***

ACE was detected in plasma and brain homogenates by measuring the amount of hyppuric acid (HA) cleaved by ACE itself from the substrate N-hyppuryl-L-histidyl-L-leucine (HHL). HA was determined by RP-HPLC according to the method already described [13] with slight modifications, as detailed below.

*Reagents preparation for HPLC analysis*

HHL (6.5 mM) and ACE (0.1 mU/ml) were dissolved in 0.1 M borate buffer containing NaCl 0.3 M at pH 8.3. HA and Captopril were dissolved in distilled water. Brain homogenates were diluted 1:100 with 0.1 M borate buffer added with NaCl 0.3M.

*Assay*

65 µL of each diluted sample of plasma or brain homogenate were preincubated with 25 µl of ACE for 10 min at 37 °C. Then 40 µl of HHL was added and the sample further incubated for 30 min at 37 °C. Enzyme reaction was terminated by adding 85 μL of HCl 1 M. Blank sample was obtained replacing sample of plasma or brain homogenates with borate buffer. Before RP-HPLC analysis, all samples were centrifuged at 5,000 rpm for 20 min and filtered.

*HPLC analysis conditions*

HA was quantified by an Agilent 1260 Infinity series liquid chromatograph system (Agilent Technologies, Palo Alto U.S.A.), including a vacuum solvent degassing unit, a binary high-pressure gradient pump, an auto sampler and an UV-DAD detector.

Chromatographic separation was performed on LC-18 column (Phenomenex Ultracarb, 4.6 x 150 mm, 5 μm). The column was eluted by a mixture of ACN (25%) and H_2_O (0.05% CH_3_COOH) (75%) at a flow rate of 0.8 mL/min. The elution was monitored at 228 nm to detect HHL and HA.

*Analysis of data*

ACE quantification was determined by a standard calibration curve prepared with a set of standard solutions containing known amount of ACE. Incubation of plasma or brain homogenates with the ACE inhibitor captopril reduced HA production by more than 94%, thus supporting the specificity of the assay.

***Determination of ACE inhibitory activity***

ACE inhibitory activity was determined according to the method already described [13] with slight modifications, as detailed below.

*Reagents preparation for HPLC analysis*

HHL (6.5 mM) and ACE (0.1 mU/ml) were dissolved in 0.1 M borate buffer containing NaCl 0.3 M at pH 8.3. Plasma was diluted 1:100 with 0.1 M borate buffer added with NaCl 0.3M.

*Assay*

65 µL of each diluted sample of plasma or brain homogenate were preincubated with 25 µl of ACE for 10 min at 37 °C. Then 40 µl of HHL was added and the sample further incubated for 30 min at 37 °C. Enzyme reaction was terminated by adding 85 μL of HCl 1 M. Blank sample was obtained replacing sample of plasma or brain homogenates with borate buffer. Before RP-HPLC analysis, all samples were centrifuged at 5,000 rpm for 20 min and filtered. HA was quantified with above reported method.

*Analysis of data*

ACE inhibitory activity was calculated from the following equation: ACE inhibition (%) = (1- Ainhibitor/Ablank) x 100, where Ainhibitor is the peak area of HA produced in presence of plasma or brain homogenates samples, Ablank is the peak area of HA in the blank sample [13].
