# Supplementary material for: Antihypertensive, cardio- and neuro-protective effects of Tenebrio molitor (Coleoptera: Tenebrionidae) defatted larvae in spontaneously hypertensive rats
Source: PLoS One. 2020 May 29;15(5):e0233788. doi: 10.1371/journal.pone.0233788 (PMC7259609; doi:10.1371/journal.pone.0233788)
Supplement: S4 File — After the sacrifice the livers were perfused in situ through the vena cava with ice-cold physiological saline solution, excised and weighed, chopped, suspended in 4 volumes of 0.25 M sucrose and homogenized in a Potter-Elvejhem homogeniser fitted with a teflon pestle. Homogenates were then centrifuged (10,000 g for 20 min) and the resulting supernatants were further centrifuged at 105,000 g for 1 h. The resulting microsomal pellets were suspended in 0.01 M Tris-HCl buffer, pH 7.6, containing 151 mM KCl, 1 mM EDTA and 20% glycerol, and stored in liquid nitrogen until use. (DOCX) [file pone.0233788.s004.docx]

**Supporting Information**

**S4 File. Liver cytochrome P450- and b5-content, NADPH-cytochrome P450 reductase activity: preparation of liver microsomes**. After the sacrifice the livers were perfused *in situ* through the vena cava with ice-cold physiological saline solution, excised and weighed, chopped, suspended in 4 volumes of 0.25 M sucrose and homogenized in a Potter-Elvejhem homogeniser fitted with a teflon pestle. Homogenates were then centrifuged (10,000 g for 20 min) and the resulting supernatants were further centrifuged at 105,000 g for 1 h. The resulting microsomal pellets were suspended in 0.01 M Tris-HCl buffer, pH 7.6, containing 151 mM KCl, 1 mM EDTA and 20% glycerol, and stored in liquid nitrogen until use.
