# Supplementary material for: Antihypertensive, cardio- and neuro-protective effects of Tenebrio molitor (Coleoptera: Tenebrionidae) defatted larvae in spontaneously hypertensive rats
Source: PLoS One. 2020 May 29;15(5):e0233788. doi: 10.1371/journal.pone.0233788 (PMC7259609; doi:10.1371/journal.pone.0233788)
Supplement: S5 File — Half brain hemispheres were quickly frozen in liquid nitrogen and stored at -80°C until analysis. The day of the assay, brains were thawed, homogenated in cold phosphate buffer 0.1 M pH 7.4 (tissue weigh: buffer volume = 1:10) and centrifuged at 10,000 g x 10 min at 4°C. Afterward the supernatant was collected and used for the ELISA assay, whose sensitivity and assay range were as follows: TNF-α sensitivity < 1 pg/ml, assay range: 7.8–500 pg/ml; IL-6: sensitivity 5.3 pg/ml, assay range 18.8–1200 pg/ml; IL-1ß sensitivity < 12 pg/ml, assay range: 25.6–2500 pg/ml. (DOCX) [file pone.0233788.s005.docx]

**Supporting Information**

**S5 File**. **Brain homogenate preparation for cytokine assay.** Half brain hemispheres were quickly frozen in liquid nitrogen and stored at -80°C until analysis. The day of the assay, brains were thawed, homogenated in cold phosphate buffer 0.1 M pH 7.4 (tissue weigh: buffer volume=1:10) and centrifuged at 10,000 g x 10 min at 4°C. Afterward the supernatant was collected and used for the ELISA assay, whose sensitivity and assay range were as follows: TNF-α sensitivity < 1 pg/ml, assay range: 7.8-500 pg/ml; IL-6: sensitivity 5.3 pg/ml, assay range 18.8-1200 pg/ml; IL-1ß sensitivity < 12 pg/ml, assay range: 25.6-2500 pg/ml.
