# Supplementary material for: Antihypertensive, cardio- and neuro-protective effects of Tenebrio molitor (Coleoptera: Tenebrionidae) defatted larvae in spontaneously hypertensive rats
Source: PLoS One. 2020 May 29;15(5):e0233788. doi: 10.1371/journal.pone.0233788 (PMC7259609; doi:10.1371/journal.pone.0233788)
Supplement: S6 File — Vascular reactivity was evaluated on aortic rings by acetylcholine and sodium nitroprusside relaxations. Briefly, the thoracic aorta was cleaned of adherent fat and rings 2.5 mm long were cut and placed between stainless-steel hooks for isometric tension recording in organ chambers. Contractile tension was recorded with a digital PowerLab data acquisition system (PowerLab 8/30; ADInstruments, Castle Hill, Australia) and analysed by using LabChart 7.3.7 Pro (Power Lab; ADInstruments). Rings were stretched to 1 g of tension and equilibrated for 1 h. After pre-contraction with phenylephrine (0.3 μM) the presence of endothelium was verified by the ability of acetylcholine (ACh, 10 μM) to induce relaxation. Concentration-response curves of aortic rings with endothelium to ACh (100 pM–10 μM) and sodium nitroprusside (SNP, 100 pM–100 μM) were performed with and without indomethacin (5 μM; 30 min). In these experiments, the vasodilator responses to ACh and SNP were expressed as percentages of phenylephrine contraction. (DOCX) [file pone.0233788.s006.docx]

**Supporting Information**

**S6 File. Vascular functionality of aorta rings.** Vascular reactivity was evaluated on aortic rings by acetylcholine and sodium nitroprusside relaxations. Briefly, the thoracic aorta was cleaned of adherent fat and rings 2.5 mm long were cut and placed between stainless-steel hooks for isometric tension recording in organ chambers. Contractile tension was recorded with a digital PowerLab data acquisition system (PowerLab 8/30; ADInstruments, Castle Hill, Australia) and analysed by using LabChart 7.3.7 Pro (Power Lab; ADInstruments). Rings were stretched to 1 g of tension and equilibrated for 1 h. After pre-contraction with phenylephrine (0.3 µM) the presence of endothelium was verified by the ability of acetylcholine (ACh, 10 µM) to induce relaxation. Concentration-response curves of aortic rings with endothelium to ACh (100 pM–10 µM) and sodium nitroprusside (SNP, 100 pM–100 µM) were performed with and without indomethacin (5 µM; 30 min). In these experiments, the vasodilator responses to ACh and SNP were expressed as percentages of phenylephrine contraction.
