# Supplementary material for: Antihypertensive, cardio- and neuro-protective effects of Tenebrio molitor (Coleoptera: Tenebrionidae) defatted larvae in spontaneously hypertensive rats
Source: PLoS One. 2020 May 29;15(5):e0233788. doi: 10.1371/journal.pone.0233788 (PMC7259609; doi:10.1371/journal.pone.0233788)
Supplement: S7 File — Spontaneously beating hearts were rapidly explanted and mounted on a Langendorff apparatus for retrograde perfusion via the aorta at a constant flow rate of 10 ml/min with a physiological salt solution (PSS) of the following composition (mM): NaCl 118, KCl 4.7, CaCl2 2.5, MgSO4 1.2, NaHCO3 25, KH2PO4 1.2, glucose 11.5, Na pyruvate 2, and EDTA 0.5, continuously bubbled with a 95% O2−5% CO2 gas mixture (pH 7.4), and kept at 37°C [53–55]. The presence of the Ca2+ buffer EDTA (0.5 mM) in PSS assures a constant and controlled free Ca2+ concentration of 2 mM (EQCAL for Windows software, Biosoft, Cambridge, UK) throughout the experimental session. (DOCX) [file pone.0233788.s007.docx]

**Supporting Information**

**S7 File. Isolated heart preparation and perfusion** Spontaneously beating hearts were rapidly explanted and mounted on a Langendorff apparatus for retrograde perfusion *via* the aorta at a constant flow rate of 10 ml/min with a physiological salt solution (PSS) of the following composition (mM): NaCl 118, [KCl](https://www.sciencedirect.com/topics/pharmacology-toxicology-and-pharmaceutical-science/potassium-chloride) 4.7, CaCl_2_ 2.5, MgSO_4_ 1.2, NaHCO_3_ 25, KH_2_PO_4_ 1.2, glucose 11.5, Na pyruvate 2, and [EDTA](https://www.sciencedirect.com/topics/pharmacology-toxicology-and-pharmaceutical-science/ethylenediaminetetraacetic-acid) 0.5, continuously bubbled with a 95% O_2_–5% CO_2_ gas mixture (pH 7.4), and kept at 37 °C [53-55]. The presence of the Ca^2+^ buffer EDTA (0.5 mM) in PSS assures a constant and controlled free Ca^2+^ concentration of 2 mM (EQCAL for Windows software, Biosoft, Cambridge, UK) throughout the experimental session.
