# Supplementary material for: Antihypertensive, cardio- and neuro-protective effects of Tenebrio molitor (Coleoptera: Tenebrionidae) defatted larvae in spontaneously hypertensive rats
Source: PLoS One. 2020 May 29;15(5):e0233788. doi: 10.1371/journal.pone.0233788 (PMC7259609; doi:10.1371/journal.pone.0233788)
Supplement: S8 File — The preparation of cortical slices were performed according to standard experimental protocols already reported [18–20]. In particular, the brain was quickly washed in artificial cerebrospinal fluid (ACSF) (composition in mM: 120 NaCl; 2 KCl; 1 CaCl2; 1 MgSO4; 25 HEPES; 1 KH2PO4; 10 glucose, pH 7.4 and previously bubbled with a 95% O2−5% CO2 gas mixture for 20–30 min) and divided into the two hemispheres. One was used for cytokine assessment, while the other was dissected and cut into 400 μm-thickness cortex slices by using a manual chopper (Stoelting Co., Wood Dale, IL). Slices were transferred into sterile 24-well culture plates containing 0.5 ml ACSF/well previously filtered by passage through a 0.2 μm sterile filter and left at room temperature (25°C) for 60 min to recover from slicing trauma (equilibration phase). During this period, the medium was removed and replaced with fresh, oxygenated filtered ACSF every 15 min (equilibration phase). After the equilibration phase, oxidative stress was induced by treating the tissue with 10 mM H2O2 for 1 h [56]. At the end of the treatment, the colorimetric MTT method was used to assess tissue viability as already reported [19]. Slice viability was expressed as a percentage of untreated slices (Basal). Tissue water gain, taken as an index of tissue edema was also assessed and it was calculated by using the formula TE = (wet weight–dry weight)(dry weight)−1 and given as g H2O (g dw)−1 [20]. (DOCX) [file pone.0233788.s008.docx]

**Supporting Information**

**S8 File**. **Preparation of brain cortical slices.** The preparation of cortical slices were performed according to standard experimental protocols already reported [18-20]. In particular, the brain was quickly washed in artificial cerebrospinal fluid (ACSF) (composition in mM: 120 NaCl; 2 KCl; 1 CaCl_2_; 1 MgSO_4_; 25 HEPES; 1 KH_2_PO_4_; 10 glucose, pH 7.4 and previously bubbled with a 95% O_2_–5% CO_2_ gas mixture for 20–30 min) and divided into the two hemispheres. One was used for cytokine assessment, while the other was dissected and cut into 400 µm-thickness cortex slices by using a manual chopper (Stoelting Co., Wood Dale, IL). Slices were transferred into sterile 24-well culture plates containing 0.5 ml ACSF/well previously filtered by passage through a 0.2 μm sterile filter, and left at room temperature (25 °C) for 60 min to recover from slicing trauma (equilibration phase). During this period, the medium was removed and replaced with fresh, oxygenated filtered ACSF every 15 min (equilibration phase). After the equilibration phase, oxidative stress was induced by treating the tissue with 10 mM H_2_O_2_ for 1 h [56]. At the end of the treatment, the colorimetric MTT method was used to assess tissue viability as already reported [19]. Slice viability was expressed as a percentage of untreated slices (Basal). Tissue water gain, taken as an index of tissue edema was also assessed and it was calculated by using the formula TE = (wet weight – dry weight)(dry weight)^−1^ and given as g H_2_O (g dw)^−1^ [20].
