# Supplementary material for: Antihypertensive, cardio- and neuro-protective effects of Tenebrio molitor (Coleoptera: Tenebrionidae) defatted larvae in spontaneously hypertensive rats
Source: PLoS One. 2020 May 29;15(5):e0233788. doi: 10.1371/journal.pone.0233788 (PMC7259609; doi:10.1371/journal.pone.0233788)
Supplement: S1 Table — (DOCX) [file pone.0233788.s009.docx]

# Supporting Information

**S1 Table.** **Diets composition and ingredients used in the present study**

| **% of** | **Standard and Captopril^a^ diet** | ***Tenebrio molitor* diet** |
| --- | --- | --- |
| Crude protein | 22.00 | 22.00 |
| Crude oils and fat | 3.5 | 5.5 |
| Crude fibres | 4.5 | 4.5 |
| Crude ash | 7.5 | 6.5 |
| Ingredients | fish meal | larvae meal^b^ |
|  | Soybean meal extracted toasted, wheat, maize, lucerne meal, whey powder, mineral dicalcium phosphate, soybean oil, corn gluten free, sodium chloride, calcium carbonate, yeast, magnesium oxide | |

^a^ Captopril content 100 mg/kg. ^b^ Obtained as reported in experimental. Diets, were realized by Mucedola S.r.l. (Settimo Milanese, Mi, Italy) and analysed using official methods such as AOAC [(Association of Official Analytical Chemists), Official Methods of Analyses of Associationof Analytical Chemist. 15th ed. AOAC, Washington, DC.; 1990].
