# Supplementary material for: Antihypertensive, cardio- and neuro-protective effects of Tenebrio molitor (Coleoptera: Tenebrionidae) defatted larvae in spontaneously hypertensive rats
Source: PLoS One. 2020 May 29;15(5):e0233788. doi: 10.1371/journal.pone.0233788 (PMC7259609; doi:10.1371/journal.pone.0233788)
Supplement: S2 Table — (DOCX) [file pone.0233788.s010.docx]

**Supporting Information**

**S2 Table.** **Effects of the feeding with standard laboratory rodent chow (SD), or SD supplemented with either TM or captopril (C) for 4 weeks on body weight**

| ***Strain*** | ***Diet*** | ***Weight (g) at week*** | | | | | ***Gain (g)*** |
| --- | --- | --- | --- | --- | --- | --- | --- |
|  |  | *0* | *1* | *2* | *3* | *4* |  |
| **WKY** | Standard | 271.5±7.1 | 291.7±6.4 | 302.0±4.6 | 311.2±4.1 | 317.8±5.7 | 46.3±2.4 |
|  | *T. molitor* | 276.0±7.0 | 287.1±7.0 | 298.7±5.0 | 310.4±5.6 | 323.0±8.1 | 47.4±4.0 |
|  | Captopril | 268.4±8.8 | 282.8±8.6 | 294.2±6.1 | 304.2±7.0 | 311.4±9.7 | 43.0±2.0 |
| **SHR** | Standard | 264.8±3.5 | 277.0±3.6 | 289.6±4.3 | 299.9±3.0 | 307.1±3.7 | 42.4±2.8 |
|  | *T. molitor* | 266.1±6.2 | 274.0±7.7 | 285.4±7.2 | 291.3±6.6 | 300.9±8.3 | 35.7±3.1 |
|  | Captopril | 267.0±3.8 | 281.9±5.5 | 308.5±5.4 | 303.5±5.4 | 312.1±7.6 | 45.1±4.3 |

Data are reported as mean±SEM.
