# Supplementary material for: Antihypertensive, cardio- and neuro-protective effects of Tenebrio molitor (Coleoptera: Tenebrionidae) defatted larvae in spontaneously hypertensive rats
Source: PLoS One. 2020 May 29;15(5):e0233788. doi: 10.1371/journal.pone.0233788 (PMC7259609; doi:10.1371/journal.pone.0233788)
Supplement: S3 Table — (DOCX) [file pone.0233788.s011.docx]

**Supporting Information**

**S3 Table. Effects of the feeding with standard laboratory rodent chow (SD), or SD supplemented with either TM or captopril (C) for 4 weeks on food intake**

| ***Strain*** | ***Diet*** | ***Daily food (g)/rat***  ***week 1-4*** | ***Strain*** | ***Diet*** | ***Daily food (g)/rat week 1-4*** |
| --- | --- | --- | --- | --- | --- |
| **WKY** | Standard | 17.68 ±0.14 | **SHR** | Standard | 18.81± 0.70 |
|  | *T. molitor* | 19.34± 1.67 |  | *T. molitor* | 17.97± 0.42 |
|  | Captopril | 17.84± 0.60 |  | Captopril | 19.61± 0.55 |

Data are reported as mean±SEM. Pre-weighed food was provided in standard stainless steel hoppers. Two-three times/week the amount of left food, including that on the bottom of the cage or spilled onto plastic sheets under the cage, was measured. Intake was calculated as the weight of food provided minus that recovered, divided for the number of rat in the cage (generally 2-4).
