# Supplementary material for: Antihypertensive, cardio- and neuro-protective effects of Tenebrio molitor (Coleoptera: Tenebrionidae) defatted larvae in spontaneously hypertensive rats
Source: PLoS One. 2020 May 29;15(5):e0233788. doi: 10.1371/journal.pone.0233788 (PMC7259609; doi:10.1371/journal.pone.0233788)
Supplement: S4 Table — (DOCX) [file pone.0233788.s012.docx]

**Supporting Information**

**S4 Table.** **Effects of the feeding for 4 weeks with standard laboratory rodent chow (SD) or SD supplemented with either Tenebrio molitor (TM) or captopril (C) on plasma total low molecular mass thiols (sum of thiol and disulphide forms) in WKY e SHR rats**

|  | **Diet** | **tCys** | **tCysGly** | **t-γGluCys** | **tGSH** |
| --- | --- | --- | --- | --- | --- |
| **WKY** | SD | 212±10 | 1.32±0.09 | 2.54±0.14 | 39.9±1.4 |
|  | TM | 218±7 | 1.29±0.14 | 2.60±0.17 | 40.4±3.1 |
|  | C | 231±22 | 1.35±0.14 | 3.09±0.18 | 40.8±2.3 |
| **SHR** | SD | 234±5 | 1.47±0.20 | 3.81±0.25 | 51.5±4.5 |
|  | TM | 248±11° | 1.90±0.17° | 3.28±0.30°° | 50.4±3.8 |
|  | C | 249±7 | 1.69±0.09 | 3.46±0.16 | 47.5±2.0 |

Abbreviations: Cys, cysteine; CysGly, cysteinylglycine; γ-GluCys, γ-glutamylcysteine, GSH, glutathione. Data (mean±SEM) are reported as µM concentration. ° P<0.05, °° P<0.01 vs WKY, same diet (Student t test, unpaired data).
