# Supplementary material for: Antihypertensive, cardio- and neuro-protective effects of Tenebrio molitor (Coleoptera: Tenebrionidae) defatted larvae in spontaneously hypertensive rats
Source: PLoS One. 2020 May 29;15(5):e0233788. doi: 10.1371/journal.pone.0233788 (PMC7259609; doi:10.1371/journal.pone.0233788)
Supplement: S5 Table — (DOCX) [file pone.0233788.s013.docx]

**Supporting Information**

**S5 Table. Effects of the feeding for 4 weeks with standard laboratory rodent chow (SD) or SD supplemented with either Tenebrio molitor(TM) or captopril (C) on liver cytochrome P450- and b5-content and NADPH-cytochrome P450 reductase activity in WKY e SHR rats**.

| ***Strain*** | ***Diet*** | *CYP450^a^* | *b5^a^* | *NADPH-cyp reductase^b^* |
| --- | --- | --- | --- | --- |
| **WKY** | **SD** | 0.61 ± 0.03 | 0.57 ± 0,03 | 27.45 ± 2.90 |
|  | **TM** | 0.60 ± 0.07 | 0.52 ± 0.05 | 26.17 ± 2.57 |
|  | **C** | 0.68±0.07 | 0.34±0.13 | 22.22±6.05 |
| **SHR** | **SD** | 0.54 ± 0.04 | 0.56 ± 0.04 | 28.05 ± 2.66 |
|  | **TM** | 0.62 ± 0.10 | 0.63 ± 0.10 | 28.94 ± 2.77 |
|  | **C** | 0.58 ± 0.10 | 0.65 ± 0.10 | 30.18 ± 7.07 |

Data are reported as mean±SEM. a: nmol x mg prot^-1^; b: nmol x mg prot^-1^ x min^-1^.
