# Supplementary figures and images for: Antihypertensive, cardio- and neuro-protective effects of Tenebrio molitor (Coleoptera: Tenebrionidae) defatted larvae in spontaneously hypertensive rats
Source: PLoS One. 2020 May 29;15(5):e0233788. doi: 10.1371/journal.pone.0233788 (PMC7259609; doi:10.1371/journal.pone.0233788)

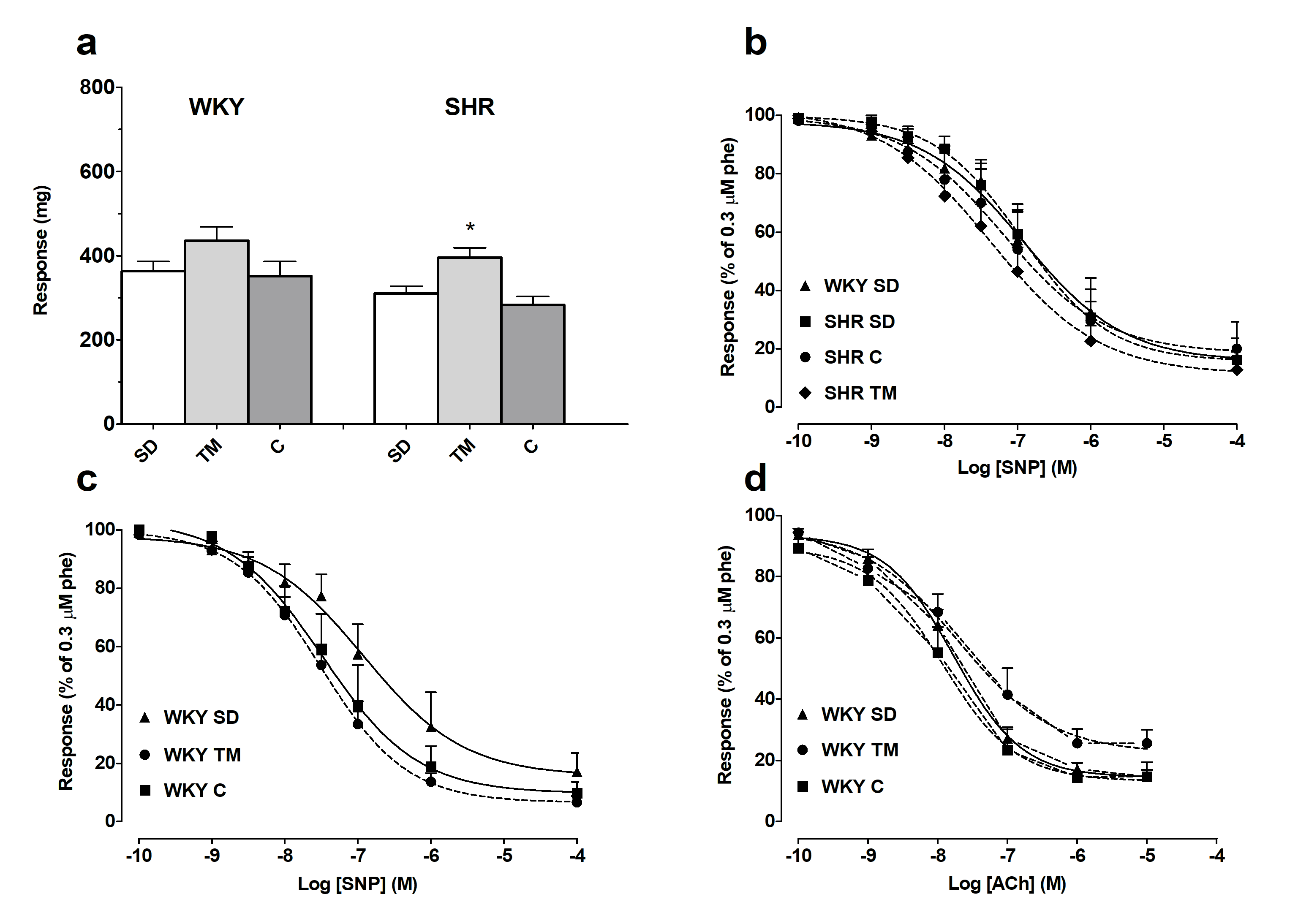

Supplement: S1 Fig — Panel a: 0.3 μM phenylephrine-induced contraction in rat aorta rings. Panel b-d: smooth muscle sensitivity to (b,c) exogenous and (d) endogenous NO: concentration-response curve to (b,c) sodium nitroprusside (SNP) of endothelium-denuded, rat aorta rings or to (d) acetylcholine (ACh) of endothelium-intact rat aorta rings pre-contracted with 0.3 μM phenylephrine. On the ordinate scale, response is reported as mg (panel a) or as percentage of the contraction induced by phenylephrine (phe), taken as 100% (panel b-d). Values are reported as mean ± SEM. *P<0.05 vs SHR SD (ANOVA followed by Dunnett post test). (TIF) [file pone.0233788.s014.tif]

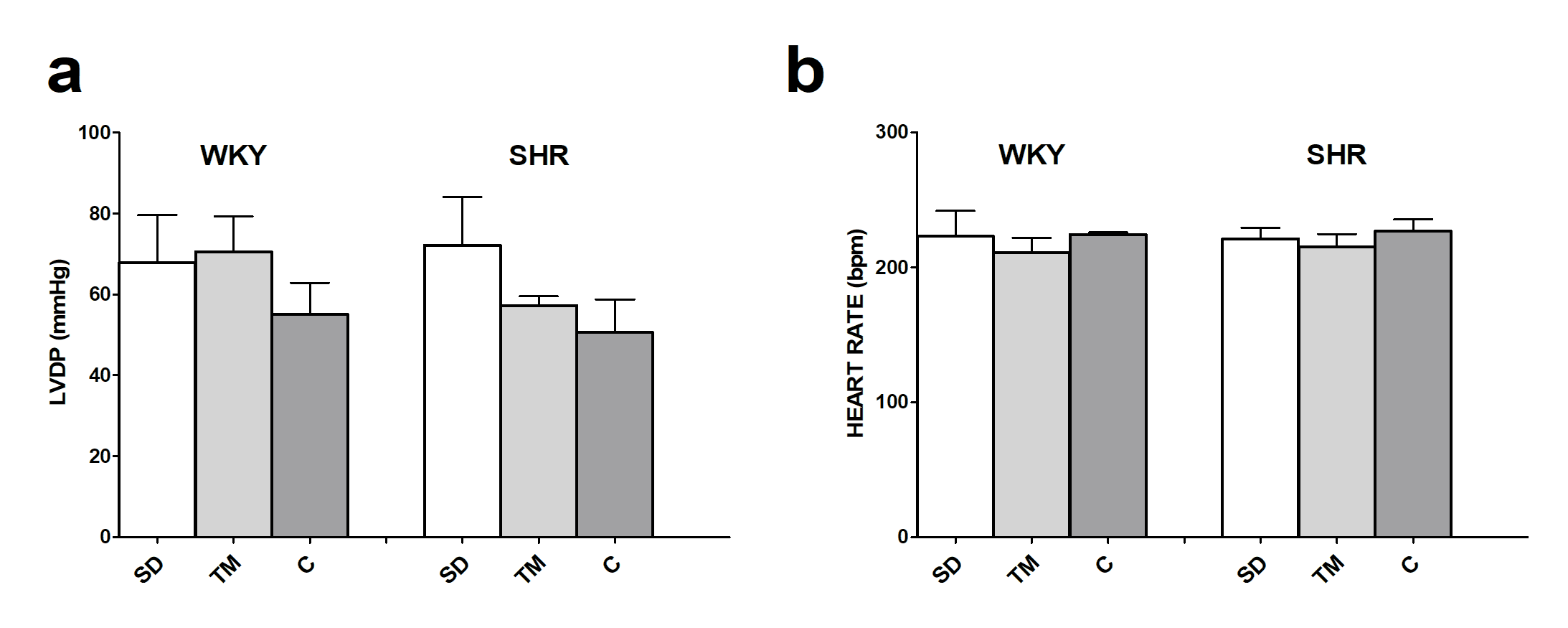

Supplement: S2 Fig — Data are reported as mean±SEM. (TIF) [file pone.0233788.s015.tif]

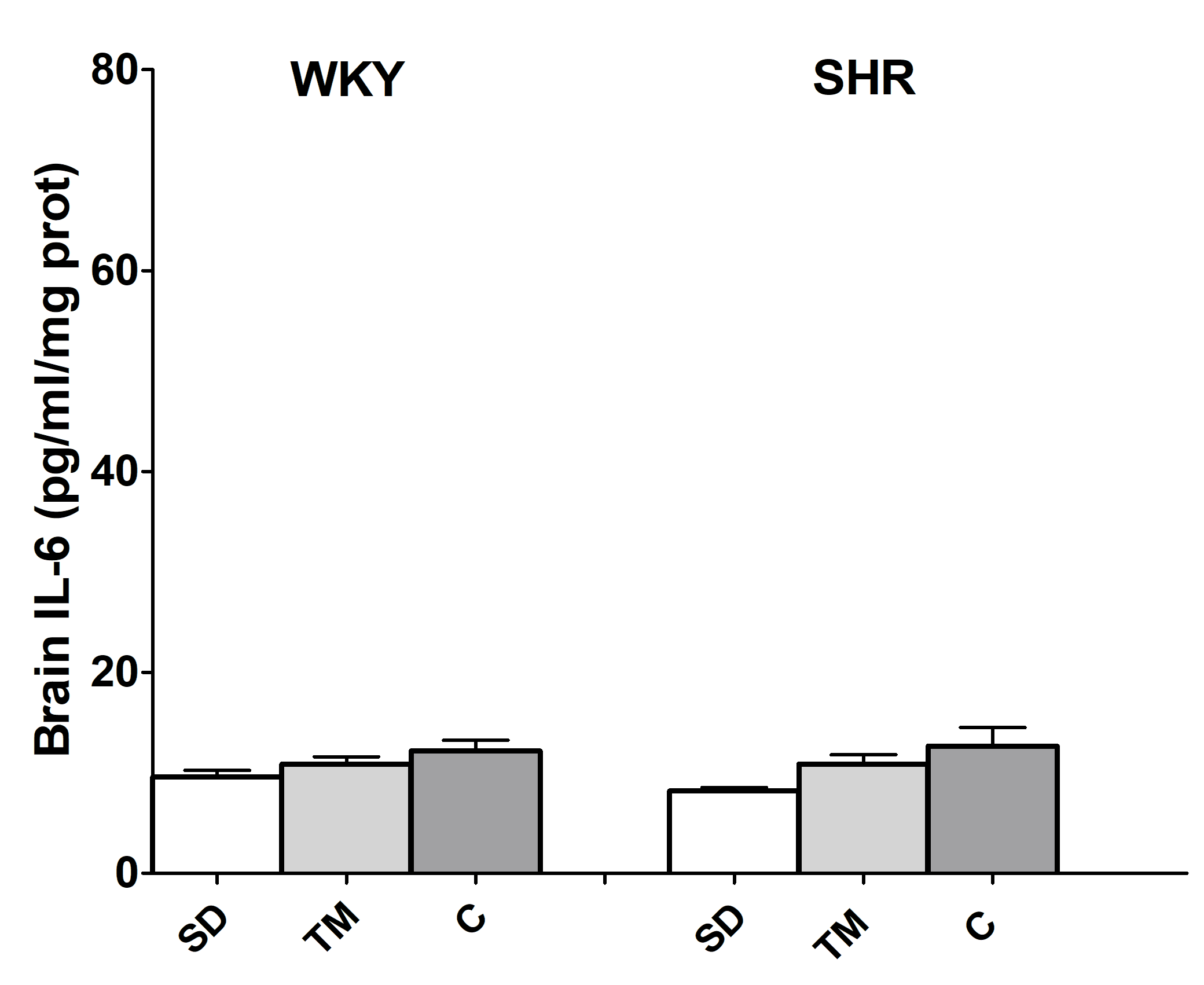

Supplement: S3 Fig — Values are reported as mean ± SEM. (TIF) [file pone.0233788.s016.tif]
