# Supplementary material for: Antihypertensive, cardio- and neuro-protective effects of Tenebrio molitor (Coleoptera: Tenebrionidae) defatted larvae in spontaneously hypertensive rats
Source: PLoS One. 2020 May 29;15(5):e0233788. doi: 10.1371/journal.pone.0233788 (PMC7259609; doi:10.1371/journal.pone.0233788)
Supplement: Attachment — Submitted filename: Rebuttal letter.docx [file pone.0233788.s018.docx]

Reviewer #1 question:
It still not clear why authors used EDTA 0.5 mM in order to buffer high Ca++ concentration .
If the idea was to use Calcium 2.0 mM, why did authors used a high concentration (2.5 mM), and them used EDTA to reduce it, as most of paper have been using extracecullar calcium in rat heart 1.2 to 1.8 mM.

As suggested by the Editor, in supporting information S7 the explanation about why EDTA was added, how free calcium was estimated were insert along with appropriate references (i.e. 53-55) about 2.5 mM CaCl2 in order to clear to the reader.
